# Network construction of gastric microbiome and organization of microbial modules associated with gastric carcinogenesis

**DOI:** 10.1038/s41598-019-48925-4

**Published:** 2019-08-27

**Authors:** Chan Hyuk Park, Jae Gon Lee, A-reum Lee, Chang Soo Eun, Dong Soo Han

**Affiliations:** Department of Internal Medicine, Hanyang University Guri Hospital, Hanyang University College of Medicine, Guri, Korea

**Keywords:** Microbiome, Gastric cancer

## Abstract

In addition to *Helicobacter pylori* infection, nitrosating/nitrate-reducing bacteria and type IV secretion system (T4SS) protein gene-contributing bacteria have been proposed as potential causes of gastric cancer development. However, bacterial modules related with gastric carcinogenesis have not been clarified. In this study, we analyzed gastric microbiome using the gastric mucosal samples obtained from the Hanyang University Gastric Microbiome Cohort by 16S rRNA gene sequencing. Weighted correlation network analysis was performed to construct a microbiome network and to identify microbial modules associated with gastric carcinogenesis. At the family level, 420 bacterial taxa were identified in the gastric microbiome of 83 participants. Through network analysis, 18 microbial modules were organized. Among them, two modules–pink and brown–were positively correlated with a higher-risk of gastric cancer development such as intestinal metaplasia with no current *H*. *pylori* infection (correlation coefficient [γ]: pink module, 0.31 [*P* = 0.004], brown module, 0.26 [*P* = 0.02]). At the family level, twenty-two and thirty-two bacterial taxa belonged to the pink and brown modules, respectively. They included nitrosating/nitrate-reducing bacteria, T4SS protein gene-contributing bacteria, and various other bacteria, including Gordoniaceae, Tsukamurellaceae, Prevotellaceae, Cellulomonadaceae, Methylococcaceae, and Procabacteriaceae. The blue module, which included *H*. *pylori*, was correlated negatively with intestinal metaplasia (γ = −0.49 [*P* < 0.001]). In conclusion, intragastric bacterial taxa associated with gastric carcinogenesis can be classified by network analysis. Microbial modules may provide an integrative view of the microbial ecology relevant to precancerous lesions in the stomach.

## Introduction

*Helicobacter pylori* infection is the most substantial risk factor for gastric carcinogenesis^1^. The International Agency for Research on Cancer has designated *H*. *pylori* as a group 1 carcinogen^2^. Chronic *H*. *pylori* infection induces chronic superficial gastritis, gastric atrophy, and intestinal metaplasia, and may cause the development of gastric cancer^3^. Additionally, various clinical traits, including age, sex, tobacco smoking, family history of gastric cancer, consumption of salty and smoked food, and low consumption of fruits and vegetables are known to be related with gastric cancer development^4^.

Recently, interest in the intragastric bacteria other than *H*. *pylori* has increased. Our group first reported the composition of the gastric microbiome depending on gastric carcinogenesis through 16 S rRNA gene sequencing^5^. We found that many bacterial taxa other than *H*. *pylori* are present in the stomach. Among non-*H*. *pylori* bacteria, nitrosating or nitrate-reducing bacteria, including *Neisseria*, *Clostridium*, and *Staphylococcus* have been proposed as potential candidates for gastric carcinogenesis^6–8^. Additionally, we found that the type IV secretion system (T4SS) protein gene-contributing bacteria including Neisseriaceae and Rhizobiales are abundant in patients with intestinal metaplasia^9^. T4SS is an essential protein for initiation of gastric carcinogenesis via transferring CagA into the gastric epithelium^10^.

However, the composition of the gastric microbiome in patients who have precancerous lesions including intestinal metaplasia remains unclear. It is possible that more unidentified bacterial taxa, as well as some known bacterial taxa, interact with each other in gastric carcinogenesis. In the current study, therefore, we evaluated the gastric microbiome associated with the gastric carcinogenesis. The advanced stage of gastric carcinogenesis was assessed using the ABCD method, which is an established gastric cancer risk assessment tool based on *H*. *pylori* infection and atrophy/intestinal metaplasia^11,12^. In the ABCD method, groups A, B, C, and D represent low, intermediate, high, and very high risk of gastric cancer, respectively. We then constructed a gastric microbiome network by weighted correlation network analysis to provide hierarchical clustering on a correlation network^13^. Through network analysis, several modules consisting of potential bacterial taxa, which correlated with gastric carcinogenesis, were organized.

## Results

### Baseline characteristics and microbiome reads

**Figure 1:**
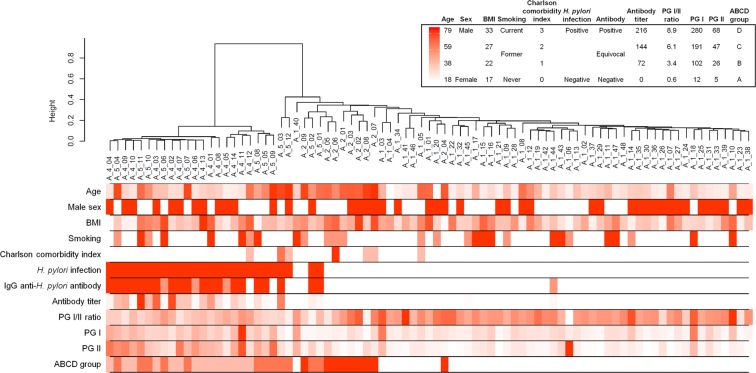
Sample dendrogram and trait heatmap. The dendrogram plotted by hierarchical clustering for gastric microbiome composition in 83 included participants. The heatmap presented below the dendrogram indicates clinical traits for the corresponding participants. ABCD group indicates categorization by *H*. *pylori* infection and atrophic gastritis as follows: (1) Group A: no *H*. *pylori* infection and no atrophic gastritis, (2) Group B: *H*. *pylori* infection and no atrophic gastritis, (3) Group C: *H*. *pylori* infection and atrophic gastritis with intestinal metaplasia, and (4) Group D: atrophic gastritis with intestinal metaplasia and no *H*. *pylori* infection. BMI, body mass index; PG, pepsinogen.

Baseline characteristics of participants in this study and gastric microbiome reads are shown in Table 1. A total of 83 participants were included in the study. The mean age was 40 years, and the proportion of males was 47.0%. *H*. *pylori* infection was identified in 31.3% of participants. Forty-eight (57.8%), 14 (16.9%), 12 (14.5%), and nine (10.8%) participants belonged to the groups A, B, C, and D, respectively. The mean of the microbiome read count was 11,720 ± 7,798. A total of 420 bacterial taxa at the family level were identified from the gastric microbiome data. The detailed data about DNA concentration, abundance of total bacteria, and number of reads in each sample are presented in Table S1. Additionally, the sample dendrogram produced by a hierarchical clustering is shown in Fig. 1.

**Table 1:** Baseline patient characteristics and microbiome reads of samples.

Variable	Value
n	83
Age, year, mean ± SD	40.1 ± 17.5
Sex, n (%)	
Male	39 (47.0)
Female	44 (53.0)
Body mass index, kg/m^2^, mean ± SD	22.7 ± 3.7
Smoking habit, n (%)	
Never	55 (66.3)
Former	13 (15.7)
Current	15 (18.1)
Charlson comorbidity index, n (%)	
0	76 (91.6)
1	5 (6.0)
2	1 (1.2)
3	1 (1.2)
*H*. *pylori* infection, n (%)	26 (31.3)
IgG anti-*H*. *pylori* antibody, n (%)	
Negative	59 (71.1)
Equivocal	4 (4.8)
Positive	20 (24.1)
Pepsinogen testing, mean ± SD	
Pepsinogen I, ng/mL	62.2 ± 40.8
Pepsinogen II, ng/mL	17.1 ± 13.0
Pepsinogen I/II ratio	4.2 ± 1.7
ABCD group	
Group A (no *H*. *pylori* infection, no atrophic gastritis and metaplasia)	48 (57.8)
Group B (*H*. *pylori* infection, no atrophic gastritis and metaplasia)	14 (16.9)
Group C (*H*. *pylori* infection, atrophic gastritis and metaplasia)	12 (14.5)
Group D (no *H*. *pylori* infection, atrophic gastritis and metaplasia)	9 (10.8)
Microbiome reads, mean ± SD	
Read count	11719.7 ± 7798.1
OTU	233.0 ± 143.9
Chao1 estimator	133.4 ± 58.9
Shannon’s diversity index	2.96 ± 1.36
Simpson’s diversity index	0.76 ± 0.32

OTU, operational taxonomic unit; SD, standard deviation.

### Module membership identification

**Figure 2:**
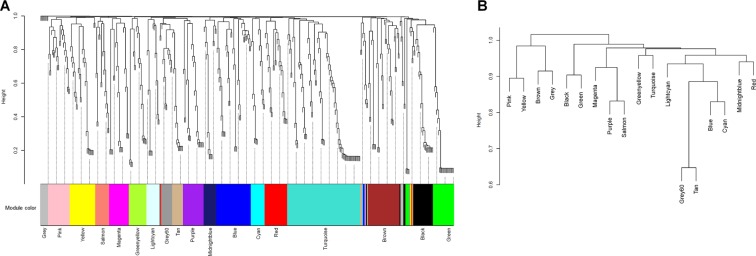
Module membership identification using dynamic tree. (**A**) Bacterial taxa dendrogram and module colors, (**B**) Clustering of module eigenvalues. In the dendrogram of panel A, each leaf, shown as a short vertical line, corresponds to a bacterial taxon. Branches of the dendrogram grouped together densely and interconnected represent highly co-occurring bacterial taxa. The highly co-occurred bacterial taxa can be classified into 18 different modules (from pink to green modules). In the clustering dendrogram of module membership, with dissimilarity based on the topological overlap, the pink, yellow, and brown modules show higher similarity to each other compared to the other modules. The gray module indicates unassigned bacterial taxa.

In Fig. 2A, highly co-occurring bacterial taxa were classified into 18 different modules (from pink to green modules). Among these 18 modules, the gray module indicated unassigned bacterial taxa. In the dendrogram, each leaf, represented as a short vertical line, corresponded to a bacterial taxon. Densely interconnected branches of the dendrogram group represented highly co-occurring bacterial taxa.

The bacterial taxa included in each module are shown in Table S2. Interestingly, most T4SS protein gene-contributing bacteria were found in either the pink or brown modules (pink module: Acidobacteriaceae, Burkholderiaceae, Neisseriaceae, and Pasteurellaceae; brown module: Bartonellaceae, Brucellaceae, unclassified Rhizobiales, Pseudomonadaceae, Sphingomonadaceae, and Xanthomonadaceae). Various nitrosating/nitrate-reducing bacteria were also identified in the pink or brown modules (pink module: Pasteurellaceae, Neisseriaceae, and Veillonellaceae; brown module: Pseudomonadaceae, Staphylococcaceae, and Xanthomonadaceae). Additionally, many other highly co-occurring bacterial taxa not mentioned above were also identified in the pink or brown modules.

In the clustering dendrogram of module membership, with dissimilarity based on the topological overlap, the pink and brown modules showed high similarity to each other compared to other modules (Fig. 2B). Additionally, module eigenvalues for the pink and brown modules were compared with respect to the ABCD group, as shown in Fig. S1. For the pink module, the gastric microbiome derived from participants of group D tended to have higher module eigenvalues than those derived from participants in other groups. Additionally, module eigenvalue for the brown module was also highest in the gastric microbiome derived from participants in group D.

As shown in Table S2, Helicobacteriaceae was included in the blue module, along with Bacteroidaceae, Clostridiaceae, and Lactobacillaceae were. The dendrogram in Fig. 2B reveals high dissimilarity between the blue module and the pink or brown modules.

### Correlation between module eigenvalue and clinical trait

**Figure 3:**
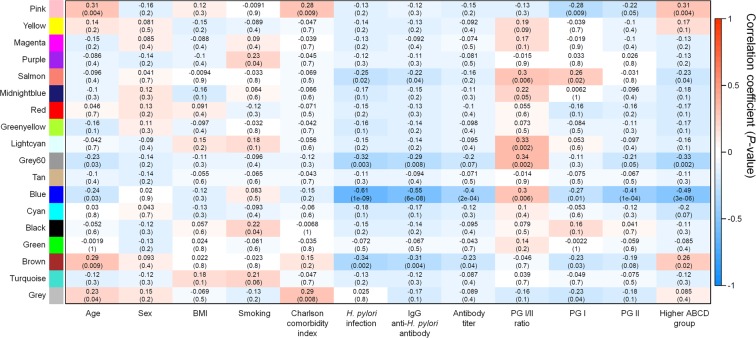
Correlation between module eigenvalue and clinical trait. Heatmap shows the correlation coefficient between module eigenvalues and clinical traits. The pink and brown modules are significantly correlated with an advanced stage of gastric carcinogenesis, or higher ABCD group (pink module, correlation coefficient [γ] = 0.31 [*P* = 0.004]; brown module, γ = 0.26 [*P* = 0.02]). These modules are also correlated with other clinical traits including age (both modules), Charlson comorbidity index (pink module only), pepsinogen I (both modules), and *H*. *pylori* infection (brown module only). ABCD group indicates categorization by *H*. *pylori* infection and atrophic gastritis as follows: (1) Group A: no *H*. *pylori* infection and no atrophic gastritis, (2) Group B: *H*. *pylori* infection and no atrophic gastritis, (3) Group C: *H*. *pylori* infection and atrophic gastritis with intestinal metaplasia, and (4) Group D: atrophic gastritis with intestinal metaplasia and no *H*. *pylori* infection, BMI, body mass index; PG, pepsinogen.

Figure 3 shows the heatmap of the correlation between module eigenvalues and clinical traits. Module eigenvalues were positively correlated with the higher ABCD group in the pink and brown groups (pink module, correlation coefficient [γ] = 0.31 [*P* = 0.004]; brown module, γ = 0.26 [*P* = 0.020]). In the yellow module also, the module eigenvalue tended to positively correlate with a higher ABCD group; however, the values were not significant. In contrast, the blue module eigenvalue was seen to negatively correlate with the higher ABCD group (γ = −0.49 [*P* < 0.001]).

In the pink module, the eigenvalues also correlated with age (γ = 0.31 [*P* = 0.004]), Charlson comorbidity index (γ = 0.28 [*P* = 0.009]), and pepsinogen I (γ = −0.28 [*P* = 0.009]). The brown module eigenvalues were seen to correlate with age (γ = 0.29 [*P* = 0.009]), *H*. *pylori* infection (γ = −0.34 [*P* = 0.002]), and pepsinogen I (γ = −0.23 [*P* = 0.030]). On the other hand, the eigenvalues of the blue module correlated with age (γ = −0.24 [*P* = 0.030]), pepsinogen I/II ratio (γ = 0.3 [*P* = 0.006], pepsinogen I (γ = −0.27 [*P* = 0.01]), and pepsinogen II (γ = −0.41 [*P* < 0.001]).

**Figure 4:**
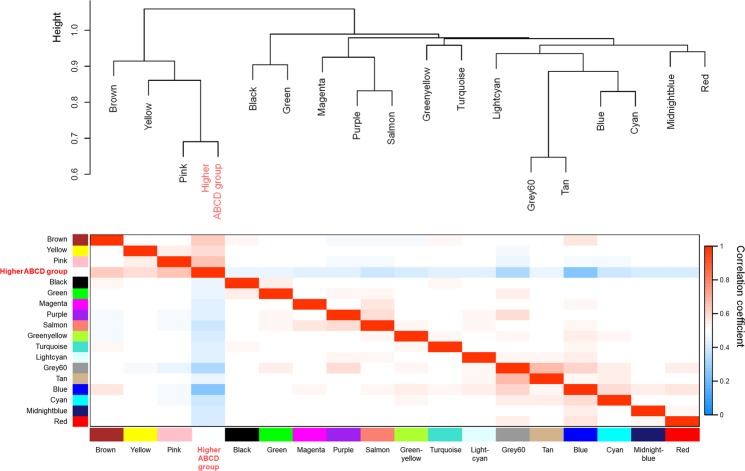
Visualization of the eigenvalue network representing relationships between the modules and ABCD groups. The dendrogram shows hierarchical clustering of the eigenvalues. In this dendrogram, the pink, yellow, and brown modules are closely related. The relationship between the higher ABCD group and the pink module is stronger than that among the pink, yellow, and brown modules. Heatmap shows the eigenvalue adjacency between modules and the higher ABCD group. The heatmap indicates that the higher ABCD group is positively correlated with the brown, yellow, and pink modules, while it is negatively correlated with the other modules. ABCD group indicates categorization by *H*. *pylori* infection and atrophic gastritis as follows: (1) Group A: no *H*. *pylori* infection and no atrophic gastritis, (2) Group B: *H*. *pylori* infection and no atrophic gastritis, (3) Group C: *H*. *pylori* infection and atrophic gastritis with intestinal metaplasia, and (4) Group D: atrophic gastritis with intestinal metaplasia and no *H*. *pylori* infection.

In Fig. 4, the eigenvalue dendrogram identifies the modules correlated with the higher ABCD groups. The dendrogram indicated that the pink, yellow, and brown modules were closely related. However, the relationship between the higher ABCD groups and the pink module was stronger than that among the pink, yellow, and brown modules. Additionally, in contrast to the pink and brown modules, the blue module was less related to the higher ABCD groups.

### Visualization of weighted networks

**Figure 5:**
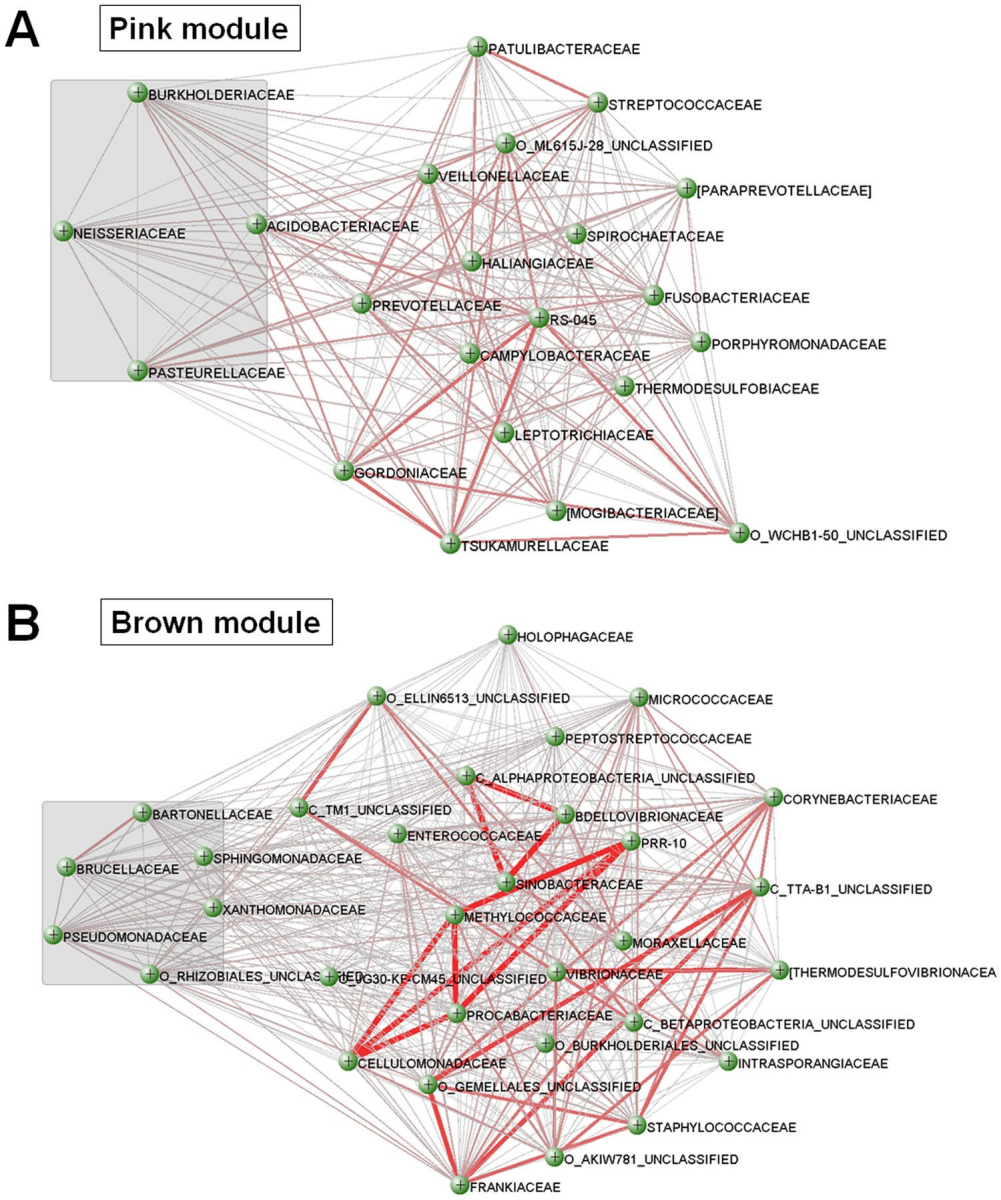
Visualization of full weighted networks in two modules associated with an advanced stage of gastric carcinogenesis. (**A**) pink module and (**B**) brown module. Strong co-occurrences among bacterial taxa other than type IV secretion system protein gene-contributing bacteria are identified in both the pink and brown modules (*i*.*e*., Gordoniaceae, Tsukamurellaceae, and Prevotellaceae in the pink module, and Cellulomonadaceae, Methylococcaceae, and Procabacteriaceae in the brown module). Gray zones represent the type IV secretion system protein gene-contributing bacteria. Thicker lines between bacterial taxa indicate high co-occurrence. Prefix “O_” and “C_” represent the order and class names, respectively. All taxa without a prefix are the family names.

Full weighted networks in the two modules associated with the higher ABCD groups, which represented an advanced stage of gastric carcinogenesis, are visualized in Fig. 5. The gray zones in the network indicate the known T4SS protein gene-contributing bacteria. Although co-occurrence among T4SS protein gene-contributing bacteria was identified in the networks, there was a stronger co-occurrence among other bacterial taxa in both the pink and brown modules (*i*.*e*., Gordoniaceae, Tsukamurellaceae, and Prevotellaceae in the pink module, and Cellulomonadaceae, Methylococcaceae, and Procabacteriaceae in the brown module). Detailed data regarding the weight of co-occurrence among bacterial taxa in all the modules are shown in Table S3.

### Validation of organized modules by network analysis with the multi-level modularity optimization method

We performed further network analysis and clustered bacterial community based on the multi-level modularity optimization method. As shown in Table S4, bacterial taxa were re-classified into 7 different modules. Most T4SS protein gene-contributing bacteria and nitrosating/nitrate-reducing bacteria that belonged to the pink or brown modules, including Bartonellaceae, Brucellaceae, Burkholderiaceae, Neisseriaceae, unclassified Rhizobiales, Pasteurellaceae, Pseudomonadaceae, and Veillonellaceae, were classified into module 1. Other T4SS protein-gene contributing bacteria and nitrosating/nitrate-reducing bacteria, including Sphingomonadaceae and Staphylococcaceae, were classified into module 2. Figure S2 shows the number of bacterial taxa re-classified into the modules 1–7 based on the multi-level modularity optimization method. Most bacterial taxa in the pink and brown modules were classified in the modules 1 and 2, respectively. The networks of the bacterial taxa in the modules 1 and 2 are presented in Fig. S3. In contrast to the pink and brown modules, bacterial taxa that belonged to the blue module were not clustered with one or two specific modules under the multi-level modularity optimization method, which indicated an inconsistent network (Fig. S2).

## Discussion

Through weighted correlation network analysis, we identified two modules associated with an advanced stage of gastric carcinogenesis (*i*.*e*., group C [intestinal metaplasia with *H*. *pylori* infection] or group D [intestinal metaplasia without *H*. *pylori* infection]), namely, the pink and brown modules. Various nitrosating/nitrate-reducing bacteria and T4SS protein gene-contributing bacteria belonged to these two modules, including the Acidobacteriaceae, Burkholderiaceae, Neisseriaceae, Pasteurellaceae, Veillonellaceae, Bartonellaceae, Brucellaceae, unclassified Rhizobiales, Pseudomonadaceae, Sphingomonadaceae, Staphylococcaceae, and Xanthomonadaceae families^6–9^. Compared to group B (*H*. *pylori* infection but no intestinal metaplasia), the abundance of *H*. *pylori* decreased and the intragastric acidity increased in group C patients. Therefore, non-*H*. *pylori* bacteria belonging to the pink or brown module can be identified in patients with group C. In group D, *H*. *pylori* almost disappears and the abundance of non-*H*. *pylori* bacteria may increase^14^. In other words, non-*H*. *pylori* bacteria belonging to the pink or brown module can be easily identified in both group C and D, rather than in group A or B. Therefore, the pink and brown modules can be correlated with both groups. Because group C and D represent higher-risk of gastric cancer development than group A and B, patients with pink- or blue-module bacteria in the stomach may be recommended to undergo surveillance endoscopy.

In this study, we identified many bacterial taxa, apart from the nitrosating/nitrate-reducing bacteria and T4SS protein gene-contributing bacteria, associated with gastric microbial networks in patients with an advanced stage of gastric carcinogenesis. These bacterial taxa included Gordoniaceae, Tsukamurellaceae, and Prevotellaceae of the pink module and Cellulomonadaceae, Methylococcaceae, and Procabacteriaceae of the brown module. Furthermore, co-occurrence among these bacteria was more prominent in the networks than that among nitrosating/nitrate-reducing bacteria or T4SS protein gene-contributing bacteria. These results suggest that much more diverse bacteria are involved in gastric carcinogenesis than previously considered.

In gastric carcinogenesis, the role of the newly identified bacterial taxa in patients with intestinal metaplasia is not fully understood. However, the potential impact of these bacteria on gastric carcinogenesis has been suggested in previous studies. For example, Gordoniaceae, Tsukamurellaceae, and Cellulomonadaceae belong to the Actinomycetales order, which is known to be predominant in patients with gastric cancer than in those with chronic gastritis^15^. Prevotella has been found not only in gastric cancer, but also in multifocal atrophic gastritis with intestinal metaplasia^16,17^. In the present study, we found high co-occurrence of these bacteria in patients with atrophic gastritis and intestinal metaplasia without *H*. *pylori* infection.

Interestingly, the blue module, which included *H*. *pylori*, was significantly distinguished from the advanced stage of gastric carcinogenesis in the dendrogram. Thus, this module might represent the gastric microbiome in patients with chronic *H*. *pylori*-associated gastritis, rather than intestinal metaplasia with *H*. *pylori* infection. The highly co-occurred bacterial taxa in the blue module, including Bacteroidaceae, Clostridiaceae, and Lactobacillaceae, may be relatively predominant in the early stage of gastric carcinogenesis (such as chronic gastritis), but not in the late stage of gastric carcinogenesis (such as atrophic gastritis with intestinal metaplasia). In a previous study, the abundance of Bacteroidales was found to be significantly decreased in patients with gastric cancer with intestinal metaplasia compared to those with non-atrophic gastritis^18^. Another study showed that *H*. *pylori* and Lactobacillus participated in co-excluding interactions in patients with intestinal metaplasia. It has also been suggested that Lactobacilli have protective effects against the neoplastic transformation of the gastric mucosa^19,20^. Although several other studies have reported that Lactobacillaceae were predominant in patients with gastric cancer compared to those with chronic gastritis^15^, these bacteria do not appear to be pathogenic in gastric carcinogenesis^21^.

However, the network of bacterial taxa in the blue module should be cautiously interpreted, because it was not reproduced in our validation analysis based on the multi-level modularity optimization method. In fact, the abundance of *H*. *pylori* dynamically changes throughout the gastric carcinogenesis^14^. Individuals who have never been infected with *H*. *pylori* (group A) have a low risk of gastric cancer and show no *H*. *pylori* in the gastric microbiome^11^. On the contrary, individuals who are infected with *H*. *pylori* (group B or C) have intermediate or high risk of gastric cancer and show *H*. *pylori* in their gastric microbiome^11^. Compared to individuals in group C, those in group B exhibit an abundance of *H*. *pylori*, but a relatively low risk of gastric cancer. In group D patients, *H*. *pylori* is not identified in the gastric microbiome, but the risk of gastric cancer is very high. These dynamic changes of *H*. *pylori* abundance may affect the inconsistent network of bacterial taxa in the blue module. However, in case of the blue module, most bacterial taxa belonging to the pink or brown modules under the weighted correlation network analysis were clustered in the same module under the network analysis with the multi-level modularity optimization method. These findings indicated that the networks in the pink or brown module are robust.

Although our study revealed the gastric microbiome networks associated with gastric carcinogenesis, there were several limitations. First, a relatively small number of patients with intestinal metaplasia were included in the study. Therefore, only bacterial taxa at the family level could be analyzed in this study because of the limited sample size. However, our analysis could identify significant co-occurred bacterial taxa related to higher ABCD groups and several microbial modules (pink and brown modules) were validated by further network analysis. Second, this was a single-center study performed in Korea, where gastric cancer is prevalent, and it may not be possible to generalize our findings worldwide. A worldwide multi-center study is, therefore, required to confirm our results. Third, although we identified several modules associated with gastric carcinogenesis, the source of bacteria in those modules has not been clarified. However, we know that gastric acids serve as a barrier to our body from external microorganisms. When the intragastric acidity decreases, oral microorganisms may enter the stomach orally and flourish. Therefore, we believe that the various intragastric microorganisms in patients with intestinal metaplasia were derived from the oral microbiome. Fourth, actual roles of the bacteria predicted to be related to gastric carcinogenesis have not yet been evaluated. Although this study aimed to construct a microbial network through 16S rRNA gene sequencing and weighted correlation network analysis, functional studies on the suggested bacteria are needed.

Despite these limitations, our study provides a better understanding of the gastric microbiome with respect to the presence of *H*. *pylori* infection and precancerous changes in the gastric mucosa. Several modules associated with an advanced stage of gastric carcinogenesis were organized by this study. These modules included nitrosating/nitrate-reducing bacteria, T4SS protein gene-contributing bacteria, and various others including Gordoniaceae, Tsukamurellaceae, Prevotellaceae, Cellulomonadaceae, Methylococcaceae, and Procabacteriaceae. Integrative view of microbial ecology based on the microbial modules in our study may help to understand microbial interactions associated with precancerous lesions in the stomach.

## Methods

### Study population

This study was planned to further analyze microbiome data obtained from the Hanyang University Gastric Microbiome Cohort. The cohort was initially established to evaluate the gastric microbial composition according to the intragastric inflammation status (KCT0001602, https://cris.nih.go.kr). Detailed data of 16S rRNA gene sequencing were reported in a previous study^9^. The Institutional Review Board on Human Subjects Research and Ethics Committee, Hanyang University Guri Hospital, Korea approved the study protocol. Informed consent was obtained from all participants. In addition, all experiments were conducted in accordance with relevant guidelines and regulations.

The Hanyang University Gastric Microbiome Cohort consisted of healthy individuals or dyspepsia patients without alarm symptoms. Endoscopic biopsy for gastric microbiome analysis, IgG anti-*H*. *pylori* antibody and pepsinogen testing were performed in all participants in the cohort. Additionally, demographic data including age, sex, weight, height, smoking habits, and comorbidities were collected. Participants who met the following criteria were excluded: (a) patients administered medications that can affect the gastric microbiome such as acid-suppressants, antacids, antibiotics, or probiotics, within 3 months prior to enrollment; (b) patients with gastric neoplasms including carcinoma, mucosa-associated lymphoid tissue lymphoma, or adenoma; and (c) patients who underwent gastrectomy.

To analyze the association between bacterial taxa and gastric carcinogenesis, each participant was classified into one of four groups based on the ABCD method as follows^11,12^: (1) Group A: no *H*. *pylori* infection and no gastric atrophy, (2) Group B: *H*. *pylori* infection and no gastric atrophy, (3) Group C: *H*. *pylori* infection and gastric atrophy with intestinal metaplasia, and (4) Group D: gastric atrophy with intestinal metaplasia and no *H*. *pylori* infection.

### Tissue acquisition and serologic testing

Four pieces of gastric mucosal tissue were endoscopically biopsied at the greater curvature side of the mid-antrum for microbiome analysis. One piece of mucosal tissue was further biopsied for the rapid urease test to evaluate the *H*. *pylori* infection. To perform histopathologic examination, one additional piece of mucosal tissue was obtained from the antrum of the stomach. It was determined that *H*. *pylori* was present when positive findings for *H*. *pylori* were found in either rapid urease test or histologic examination. For serologic assessments, IgG anti-*H*. *pylori* antibody and pepsinogen (PG) I/II tests were performed using enzyme and latex agglutination turbidimetric immunoassays, respectively.

### Extraction of DNA

The methods of DNA extraction from mucosal biopsy samples were described previously^5,9^. Briefly, 100 mg of frozen gastric samples was suspended in 750 μL sterile bacterial lysis buffer (200 mmol/L sodium chloride, 100 mmol/L ethylenediaminetetraacetic acid, 20 mmol/L Tris base, and 20 mg/mL lysozyme) and incubated at 37 °C for 30 min. Then, we added 20 μL proteinase K and 80 μL 10% sodium dodecyl sulfate to the mixture, and incubated it at 65 °C for 30 min. Bead beating was performed for 90 s at 6.9 g (PRECELLYS 24; Bertin Technologies, Le Bretonneux, France) following adding 300 mg of 0.1 mm zirconium beads (BioSpec Products, Bartlesville, OK, USA) to complete the homogenization. The homogenized mixture was cooled on ice and centrifuged at 18.3 g for 5 min. DNA was extracted from the supernatant using phenol/chloroform/isoamyl alcohol (25:24:1), then chloroform/isoamyl alcohol (24:1) and followed by precipitated with absolute ethanol at −20 °C for 1 h. The precipitated DNA was suspended in DNase-free H_2_O and cleaned using a DNA clean-up kit (QIAGEN, Hilden, Germany). We stored isolated DNA at −80 °C until microbial characterization.

### 16S rRNA gene sequencing and analysis

For identifying microbial composition, we conducted 16 S rRNA gene sequencing, as previously described^9^. The DNA quantification and quality assessment was done by PicoGreen and Nanodrop, respectively. Input gDNA was amplified with 16 S V3–V4 primers. To add multiplexing indices and Illumina sequencing adapters, limited‐cycle amplification was performed. The 16S V3–V4 primers were as follows: forward, 5′-TCGTCGGCAGCGTCAGATGTGTATAAGAGACAGCCTACGGGNGGCWGCAG-3′ and reverse, 5′-GTCTCGTGGGCTCGGAGATGTGTATAAGAGACAGGACTACHVGGGTATCTAATCC-3′. We normalized and pooled the final products using PicoGreen. The library size was verified using the TapeStation DNA screentape D1000 (Agilent). We then sequenced the DNA using the MiSeq™ platform (Illumina, San Diego, CA, USA) and the data was analyzed using QIIME version 1.9.0^22^.

Low-quality reads with incorrect primer sequences or ambiguous bases were excluded. Using the unique nucleotide barcodes, the reads were classified into groups. We normalized the read count to the corresponding copy number of 16S rRNA genes^23^. Taxonomic assignment were performed based on a 97% similarity with the GreenGenes database (version 13.5) using QIIME.

DNA sequences obtained from this study have been deposited in the National Center for Biotechnology Information short read archive under the Accession No. SRP109017.

### Module identification and network visualization

To cluster bacterial taxa, weighted correlation network analysis was performed^24,25^. First, we chose the soft thresholding power based on the criterion of approximate scale-free topology^26^. Next, hierarchical clustering was performed to produce a hierarchical clustering tree of bacterial taxa known as a dendrogram. Using the dynamic tree cut method, highly co-occurred bacterial taxa were classified into several module memberships^26^. Next, we quantified the associations of bacterial taxa with clinical factors of interest including the ABCD group. For each module, the correlation of the module eigenvalue and bacterial taxa abundance profile was demonstrated. The module eigenvalue was defined as the first principal component of the abundance matrix of the corresponding module^26^. The Eigenvalue provided a mathematically optimal method of summarizing the co-occurrence patterns of all bacterial taxa belonging to each module. Through this analysis, potential modules and bacterial taxa associated with gastric carcinogenesis were identified. Finally, we plotted the network consisting of bacterial taxa in the module associated with gastric carcinogenesis to visualize the bacterial network. In a weighted network, all taxa were connected to each other, and these connections showed continuous weight values between 0 and 1, indicating the strength of co-regulation between taxa.

To validate our module organization based on the weighted correlation network analysis, we further performed a network analysis. For this, the bacterial community was clustered based on the multi-level modularity optimization method^27^. Bacterial taxa belonging to the modules associated with the advanced gastric carcinogenesis stage derived from the weighted correlation network analysis were compared to those in the modules derived from the network analysis with the multi-level modularity optimization method.

### Statistical analysis

Continuous and categorical variables were expressed as the means ± standard deviation and numbers with proportions, respectively. The weighted correlation network analysis was performed using the statistical software R (version 3.6.0; R Foundation for Statistical Computing, Vienna, Austria) with the WGCNA package (version 1.66; Peter Langfelder). Module eigenvalues among the ABCD groups were compared using Kruskal-Wallis test. The Benjamini-Hochberg approach was used to correct the *P*-values for multiple testing. The network analysis with multi-level modularity optimization method was conducted using igraph package (version 1.2.4.1) in R. All statistical procedures were conducted using R. Visualization of a microbial network in the modules derived from the weighted correlation network analysis were performed using VisANT 5.51, which is a data-integrating visual framework for biological networks and modules^28^.

## Supplementary information

Supplementary Information

Table S1

Table S2

Table S3

Table S4
