# Supplementary material for: Network construction of gastric microbiome and organization of microbial modules associated with gastric carcinogenesis
Source: Sci Rep. 2019 Aug 27;9:12444. doi: 10.1038/s41598-019-48925-4 (PMC6712011; doi:10.1038/s41598-019-48925-4)
Supplement: Supplementary file 1 — Supplementary Information [file 41598_2019_48925_MOESM1_ESM.pdf]

Network construction of gastric microbiome and organization of microbial modules associated with gastric carcinogenesis

Chan Hyuk Park, MD, PhD, Jae Gon Lee, MD, A-reum Lee, MS, Chang Soo Eun, MD, PhD, Dong Soo Han, MD, PhD

Department of Internal Medicine, Hanyang University Guri Hospital, Hanyang University College of Medicine, Guri, Korea

Correspondence to:

Dong Soo Han, MD, PhD

Department of Internal Medicine, Hanyang University Guri Hospital, Hanyang University College of Medicine, 153 Gyeongchun-ro, Guri, 11923 Republic of Korea

Phone: +82-31-560-2226, Fax: +82-31-553-7369, E-mail: hands@hanyang.ac.kr

Supplementary information

Table S1. DNA concentration, abundance of total bacteria, and number of reads in each sample

[a]The abundance of total bacterial DNA was calculated with the $2^{-\Delta\Delta CT}$ method using GAPDH levels for normalization. These values could not be analyzed in several cases due to insufficient remnant DNA samples.

PCR, polymerase chain reaction; OTU, operational taxonomic unit; N/A, not available

Table S2. Bacterial taxa according to modules based on the weighted correlation network analysis

Table S3. Weight of co-occurrence in the weighted correlation network analysis

Table S4. Bacterial taxa according to modules based on the multi-level modularity optimization method

Figure S1. Module eigenvalues according to the ABCD group

Point indicates the module eigenvalue in each patient.

Box plot indicates the median and interquartile ranges of the module eigenvalue in each group. The eigenvalue in the pink module is higher in group D than in group B. The eigenvalue in the brown module is higher in group D than in the other groups.

ABCD group indicates categorization by *H. pylori* infection and atrophic gastritis as follows: (1) Group A: no *H. pylori* infection and no atrophic gastritis, (2) Group B: *H. pylori* infection and no atrophic gastritis, (3) Group C: *H. pylori* infection and atrophic gastritis with intestinal metaplasia, and (4) Group D: atrophic gastritis with intestinal metaplasia and no *H. pylori* infection

$^{*}P<0.05$, $^{**}P<0.01$

Figure S2. The bacterial taxa re-classified into modules 1 to 7 based on the network analysis and multi-level modularity optimization.

Most bacterial taxa in the pink and brown modules are classified in the modules 1 and 2, respectively, while bacterial taxa belonging to the blue module are not clustered with one or two specific modules under the multi-level modularity optimization method.

There is no bacterial taxon belonging to the module 7 among bacterial taxa in the pink, brown, and blue modules.

Figure S3. Network visualization of bacterial taxa belonging to the modules 1 or 2 based on the multi-level modularity optimization method.

Edge between bacterial taxa indicates the co-occurrence of bacterial taxa in the gastric microbiome.

(A) module 1, and (B) module 2

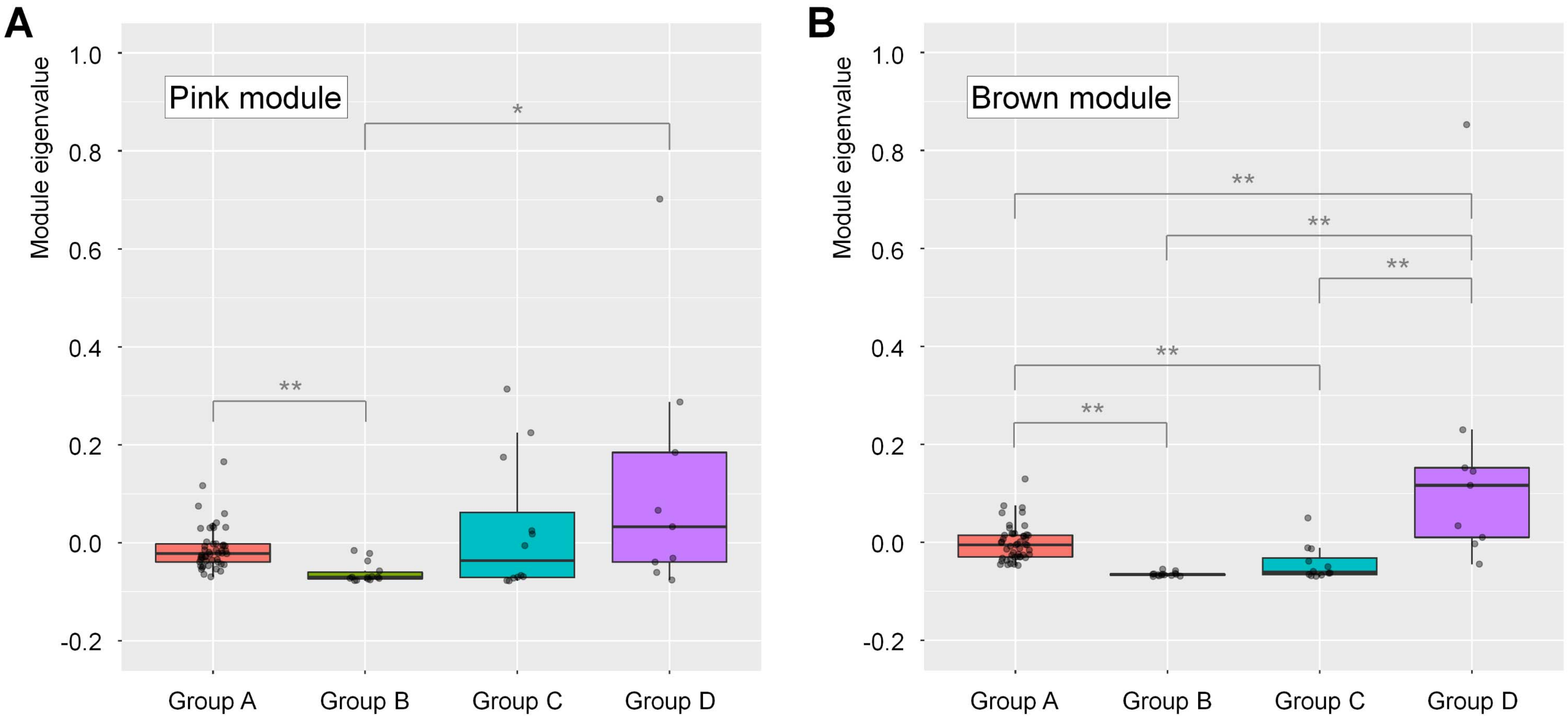

Figure S1. Module eigenvalues according to the ABCD group

Point indicates the module eigenvalue in each patient.

Box plot indicates the median and interquartile ranges of the module eigenvalue in each group. The eigenvalue in the pink module is higher in group D than in group B. The eigenvalue in the brown module is higher in group D than in the other groups.

ABCD group indicates categorization by *H. pylori* infection and atrophic gastritis as follows: (1) Group A: no *H. pylori* infection and no atrophic gastritis, (2) Group B: *H. pylori* infection and no atrophic gastritis, (3) Group C: *H. pylori* infection and atrophic gastritis with intestinal metaplasia, and (4) Group D: atrophic gastritis with intestinal metaplasia and no *H. pylori* infection

$^{*}P<0.05$, $^{**}P<0.01$

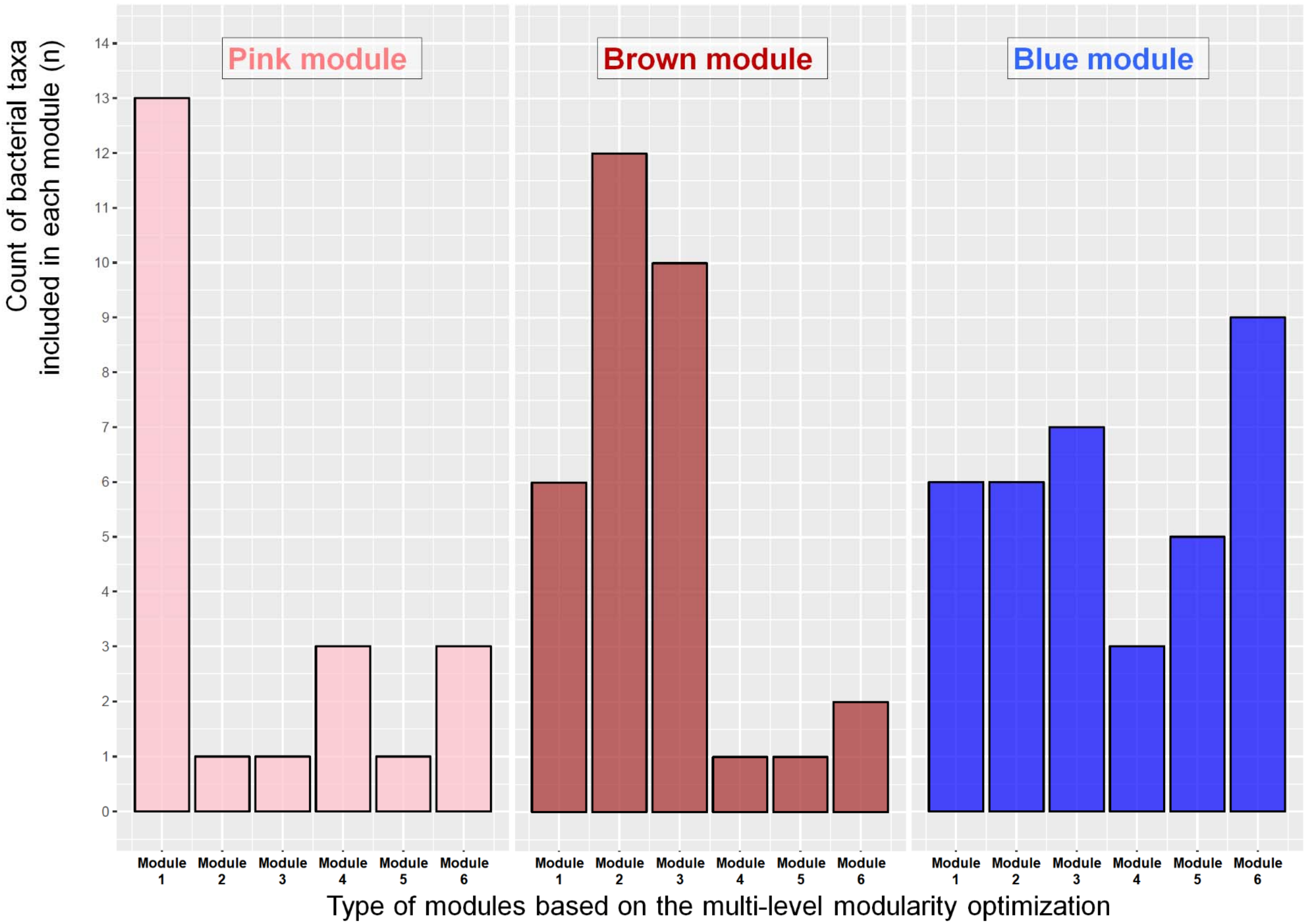

Figure S2. The bacterial taxa re-classified into modules 1 to 7 based on the network analysis and multi-level modularity optimization.

Most bacterial taxa in the pink and brown modules are classified in the modules 1 and 2, respectively, while bacterial taxa belonging to the blue module are not clustered with one or two specific modules under the multi-level modularity optimization method.

There is no bacterial taxon belonging to the module 7 among bacterial taxa in the pink, brown, and blue modules.

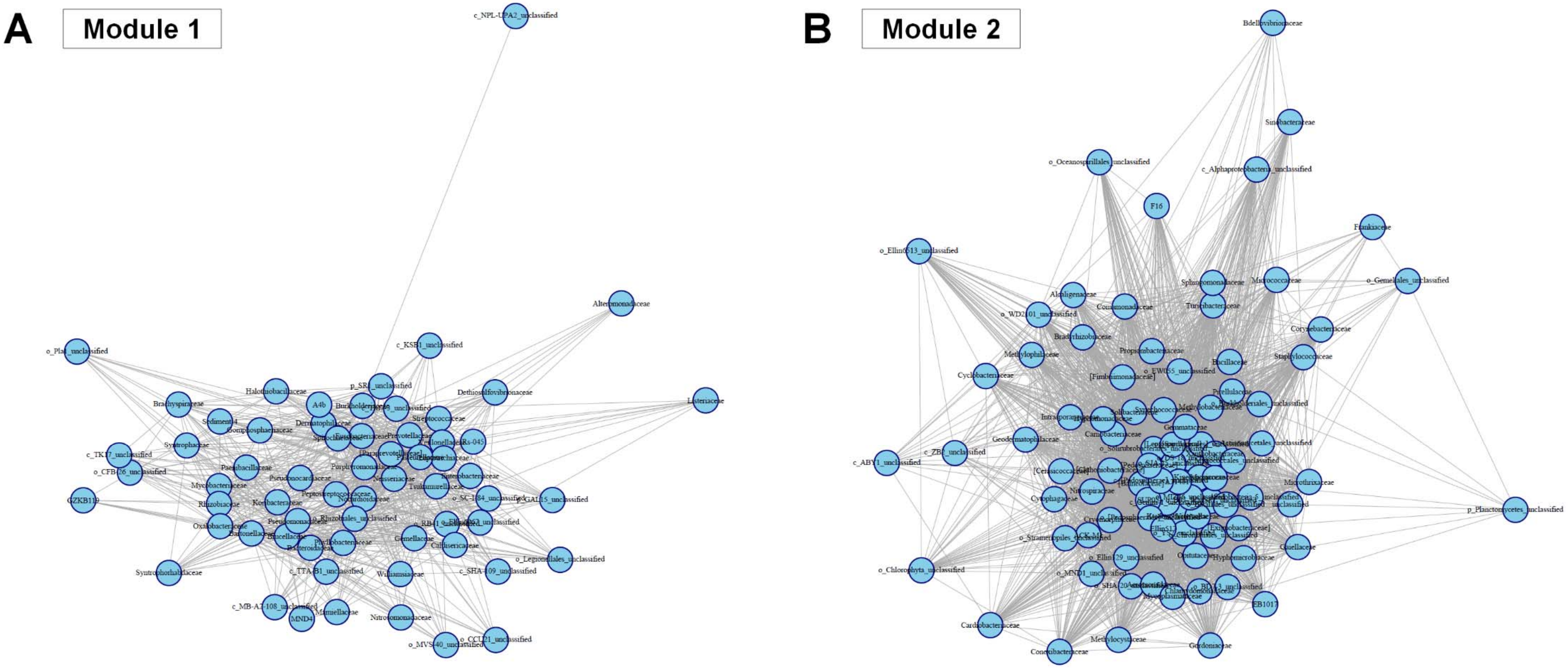

Figure S3. Network visualization of bacterial taxa belonging to the modules 1 or 2 based on the multi-level modularity optimization method.

Edge between bacterial taxa indicates the co-occurrence of bacterial taxa in the gastric microbiome.

(A) module 1, and (B) module 2
